# Calculus-targeted puncture technique to overcome access difficulty during endoscopic ultrasound-guided hepaticogastrostomy

**DOI:** 10.1055/a-2688-6469

**Published:** 2025-09-26

**Authors:** Yasuhisa Hiroshima, Ryota Sagami, Yoshifumi Azuma, Hiroaki Tsuji, Hidefumi Nishikiori, Kazuhiro Mizukami, Kazunari Murakami

**Affiliations:** 1 13235 Department of Gastroenterology, Oita University Faculty of Medicine, Yufu, Japan; 2 13235 Department of Advanced Gastrointestinal Cancer Medicine, Oita University Faculty of Medicine, Yufu, Japan; 3 157533 Department of Gastroenterology, Oita San-ai Medical Center, Oita, Japan

An 83-year-old woman was admitted to undergo endoscopic treatment for recurrent cholangitis caused by intrahepatic bile duct stones. Computed tomography revealed oval stones within the left intrahepatic bile ducts ( Fig. 1 ). Stone removal via balloon enteroscopy-assisted endoscopic retrograde cholangiopancreatography (BE-ERCP) was considered technically challenging owing to a history of three previous bile duct surgeries. Therefore, treatment with an endoscopic ultrasound (EUS)-guided technique was selected.

**Fig. 1:**
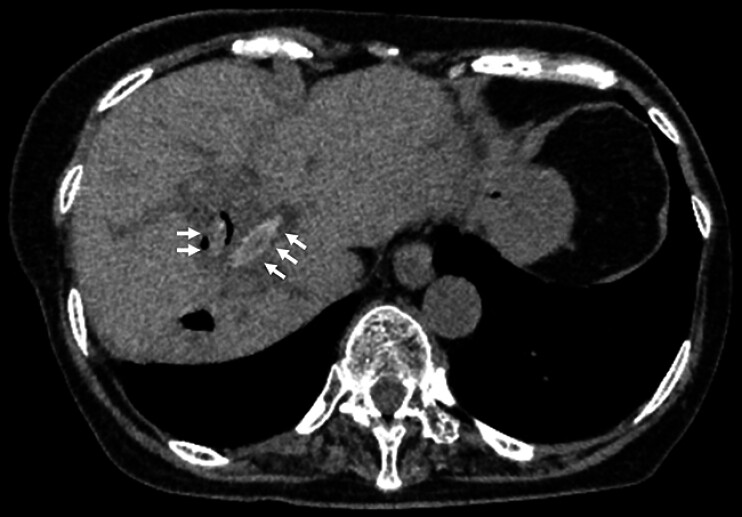
Computed tomography image showing oval stones in the left intrahepatic bile ducts (white arrows).

Initially, EUS-guided hepaticogastrostomy (EUS-HGS) was performed via the B3 bile duct Fig. 2 **a** ); however, the hepatic parenchyma was extremely thin owing to repeated inflammation, making safe puncture and stent placement unfeasible. Although the B2 duct – dilated to approximately 4 mm – was subsequently targeted, puncture failed owing to its deep location and poor alignment with the needle trajectory.

**Fig. 2:**
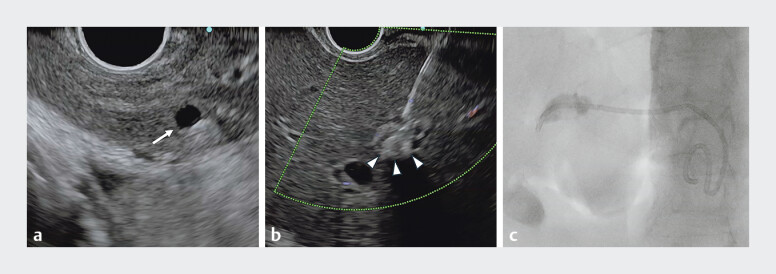
Images during endoscopic ultrasound-guided hepaticogastrostomy (EUS-HGS) showing: **a** the initial attempt via the B3 bile duct (white arrow), which failed owing to the thin hepatic parenchyma that made safe puncture and stent placement unfeasible; **b** the echogenic calculus-targeted puncture being performed, using the intrahepatic stone (arrowheads) as the puncture target; **c** a dedicated plastic stent for EUS-HGS that was successfully deployed without adverse events.

Consequently, an echogenic calculus was selected as the puncture target ( Fig. 2 **b** ). This novel technique significantly improved visualization and allowed for the establishment of a safer and more precise puncture route ( Video 1 ). Access was successfully obtained using a 19-gauge needle, and a 0.025-inch guidewire was advanced smoothly through the B2 intrahepatic duct into the intestinal lumen beyond the stones. A second guidewire was inserted using a double-lumen cannula (Uneven Double Lumen Cannula; Piolax, Kanagawa, Japan), and this was followed by successful placement of a dedicated plastic stent for EUS-HGS (TYPE-IT stent, 7 Fr, 14 cm; Gadelius Medical, Tokyo, Japan) without any adverse events ( Fig. 2 **c** ). Cholangioscopy-guided electrohydraulic lithotripsy performed 5 months later, after dilation of the endosonographically created route, achieved complete stone clearance ( Fig. 3 ).

The calculus-targeted puncture technique is used during endoscopic ultrasound-guided hepaticogastrostomy, enabling cholangioscopy-guided lithotripsy and achieving complete removal of recurrent intrahepatic bile duct stones.Video 1

**Fig. 3:**
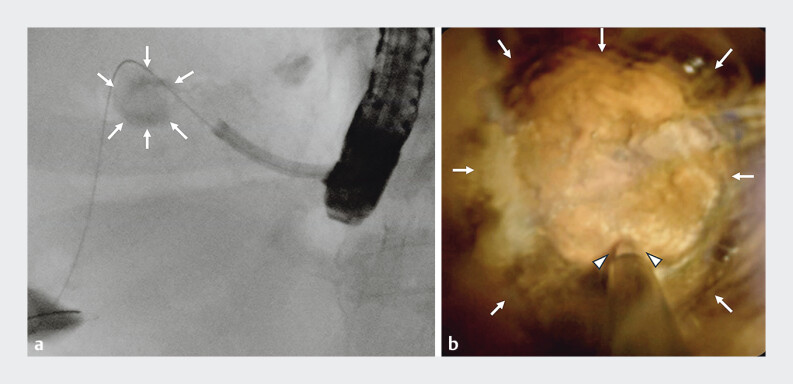
Images during stone removal showing an intrahepatic bile duct stone (white arrows) with the lithotripter (arrowheads) positioned via the endosonographically created route to perform cholangioscopy-guided electrohydraulic lithotripsy on: **a** fluoroscopic image; **b** cholangioscopic image.

In cases where BE-ERCP is challenging owing to surgically altered anatomy, EUS-guided treatment has been reported as an effective approach for stone removal 1 2 3 . For patients with intrahepatic large bile duct stones and limited access routes, this novel calculus-targeted puncture technique may enhance visualization and improve puncture precision. Further studies are warranted to validate its safety and utility.

Endoscopy_UCTN_Code_TTT_1AS_2AH

## Notes

Endoscopy E-Videos https://eref.thieme.de/e-videos
*E-Videos* is an open access online section of the journal *Endoscopy* , reporting on interesting cases and new techniques in gastroenterological endoscopy. All papers include a high-quality video and are published with a Creative Commons CC-BY license. Endoscopy E-Videos qualify for HINARI discounts and waivers and eligibility is automatically checked during the submission process. We grant 100% waivers to articles whose corresponding authors are based in Group A countries and 50% waivers to those who are based in Group B countries as classified by Research4Life (see: https://www.research4life.org/access/eligibility/ ). This section has its own submission website at https://mc.manuscriptcentral.com/e-videos .
